# Mental Health and Well-Being Needs among Non-Health Essential Workers during Recent Epidemics and Pandemics

**DOI:** 10.3390/ijerph19105961

**Published:** 2022-05-13

**Authors:** Nashit Chowdhury, Ankit Kainth, Atobrhan Godlu, Honey Abigail Farinas, Saif Sikdar, Tanvir C. Turin

**Affiliations:** 1 Department of Family Medicine, Cumming School of Medicine, University of Calgary, 3330 Hospital Dr NW, Calgary, AB T2N 4N1, Canada; nashit.chowdhury@ucalgary.ca; 2 Department of Community Health Sciences, Cumming School of Medicine, University of Calgary, Calgary, AB T2N 4N1, Canada; 3 Alberta International Medical Graduates Association, Calgary, AB T2E 3K8, Canada; mdsaifuddin.sikdar@ucalgary.ca; 4 Community Scholar and Citizen Researcher, Immigrant and Refugee Health Interest Group, Calgary, AB T2N 4N1, Canada; drankitkainth1984@gmail.com (A.K.); agodlu2@gmail.com (A.G.); abifarinas1621@yahoo.ca (H.A.F.); 5 Department of Microbiology, Immunology and Infectious Disease, Cumming School of Medicine, University of Calgary, Calgary, AB T2N 4N1, Canada

**Keywords:** mental health, essential workers, non-health essential workers, frontline workers, vulnerable population, pandemic, epidemic, outbreak

## Abstract

Essential workers, those who work in a variety of sectors that are critical to sustain the societal infrastructure, were affected both physically and mentally by the COVID-19 pandemic. While the most studied group of this population were healthcare workers, other essential non-health workers such as those working in the law enforcement sector, grocery services, food services, delivery services, and other sectors were studied less commonly. We explored both the academic (using MEDLINE, PsycInfo, CINAHL, Sociological Abstracts, and Web of Science databases) and grey literature (using Google Scholar) to identify studies on the mental health effects of the six pandemics in the last 20 years (2000–2020). We identified a total of 32 articles; all of them pertained to COVID-19 except for one about Ebola. We found there was an increase in depression, anxiety, stress, and other mental health issues among non-health essential workers. They were more worried about passing the infection on to their loved ones and often did not have adequate training, supply of personal protective equipment, and support to cope with the effects. Generally, women, people having lower education, and younger people were more likely to be affected by a pandemic. Exploring occupation-specific coping strategies of those whose mental health was affected during a pandemic using more robust methodologies such as longitudinal studies and in-depth qualitative exploration would help facilitate appropriate responses for their recovery.

## 1. Introduction

Essential workers have gained attention during the current pandemic, as they have played the most crucial roles in combating COVID-19 [1]. They have helped maintain the socioeconomic infrastructure by putting themselves at greater risk of being infected and infecting their families. Essential workers are defined as those “who conduct a range of operations and services that are typically essential to continue critical infrastructure viability” such as healthcare workers, those in the law enforcement sector, grocery services, food services, delivery services, and others [2,3]. A longitudinal survey conducted in Canada found a significant increase in stress during the COVID-19 outbreak [4,5]. Studies on health-related essential workers have been more prevalent and have reported high rates of depression, anxiety, stress, and other mental health issues in this group [6]. A few studies on non-health essential workers such as grocery workers [7] and protective services workers [8] also found an increase in mental health issues among these workers due to COVID-19.

According to the Mental Health Research Canada (MHRC) national polling initiative, anxiety and depression have increased greatly among the overall population since COVID-19 began [9]. The number of individuals with a high level of anxiety has tripled (from 8% to 24%) and the number of those with a high level of depression has doubled (7% to 15%) since the beginning of the pandemic [9]. A study involving both health and non-health (e.g., transportation, food, etc.) essential workers in Spain and Brazil found that around 8–27% of people suffered from depression, anxiety, or both [10]. In addition to healthcare workers [11], non-health essential workers such as domestic workers and police personnel were also reported to have inadequate access to personal protective equipment (PPE), resulting in increased fear and worries [12,13]. They were reported to be overworked, stigmatized, and isolated from their family members, which increased stress on their mental health [11,14,15,16]. Some studies on healthcare essential workers during previous pandemics such as severe acute respiratory syndrome (SARS) in 2002, Ebola in 2014, middle east respiratory syndrome (MERS) in 2016, and others also reported increased mental health-related effects such as depression and anxiety symptoms and insomnia, each ranging from 27–50% [17,18].

The effect of a pandemic on mental health may be influenced by many factors related to sociodemographic characteristics, workplace environment, and individual characteristics. Previous studies found gender, age, education level, income status, occupation, and support from colleagues and employers to be related to mental health symptoms [19]. Pandemics are a special situation in which different individuals from different occupations may experience the effects disproportionately. For example, people with higher education may comprehend the risks and dangers better than those with lower educations and practice healthy strategies to cope with that, whereas their counterparts may panic and adopt self-harming practices [20]. In addition, the ongoing COVID-19 pandemic has highlighted social support and health- and wellness-care-related inequities within various population groups, including essential workers.

According to a report by the American Psychological Association, both health and non-health essential workers were less likely to receive mental health treatment from professionals (34% vs. 12%) despite being almost three times more likely to be diagnosed with a mental health condition due to the pandemic (25% vs. 9%) [21]. Many reported adopting unhealthy practices such as poor eating and drinking habits [22]. According to a study involving food and retail service workers, some individuals employed potentially dangerous coping mechanisms such as consuming alcohol, smoking, and social avoidance [23]. In another US study, about 10–18% of the people who needed mental health support during this pandemic, including essential workers and the general population, did not receive any counselling or therapy [24]. Individuals with mental health issues may not access proper psychiatric support because of cost barriers or lack of time [25].

The non-health essential workforce such as grocery workers, transportation workers, food and delivery service workers, and many others have received little attention despite their selfless and unceasing efforts throughout the pandemic. Many studies, including literature reviews, have been conducted on the mental health effects of pandemics, specifically on healthcare essential workers [17,26] and general populations [27,28]. However, despite the fact that non-health essential workers may experience worse mental health symptoms/effects due to COVID-19 compared to healthcare workers and the general population, there are limited studies on this population group [29]. While non-health essential workers were at risk of contracting COVID-19 similar to that of health workers, they may not have the same level of understanding or confidence of how to stay safe from the virus because of a lack of adequate training or safety measures promoted and practiced by their employers [29]. Given the accelerated globalization, merging societal and political structures, and comparable worldwide actions against pandemics in recent decades, often guided and monitored by various international health organizations, we undertook this scoping review to capture the mental health effects of previous pandemics on non-health essential workers in the last 20 years (2000–2020). We also attempted to identify factors affecting their mental health and the coping strategies they adopted.

## 2. Methods

We opted to undertake a comprehensive scoping review for this study, as they provide a detailed snapshot of the existing literature on a certain topic and identify research gaps to offer future research directions [30]. We followed Arksey and O’Malley’s methodological framework for scoping reviews [31].

### 2.1. Formulating the Research Question

A scoping review allows us to answer a broader question by exploring studies that used diverse methodologies [32]. For this scoping review, we asked the following questions:

(1) What were the mental health effects on non-health essential workers during pandemics in the last 20 years (2000–2020)?
(2) What were the factors affecting their mental health (including those related to the onset of their mental health issues and those that aggravated and alleviated the mental health effects)?
(3) What were their coping strategies to combat the mental health effects?

### 2.2. Identifying Relevant Studies

We conducted an extensive search using appropriate keywords to determine the relevant literature for this review. We used all possible search-terms (Table 1) related to mental health, the six pandemics in the last 20 years, and essential workers. We explored both the academic and grey literature by searching the most common electronic databases for both types of articles (Table 2). The potential literature was further included by snowball sampling from the reference lists of selected articles. Different keywords for each term were connected by the Boolean operator “OR” and were later collectively linked for each main term using the Boolean operator “AND”.

### 2.3. Study Selection

A comprehensive literature search generally culminates in a large number of articles encumbered by duplicate and irrelevant studies, which requires screening vigilantly by applying clearly defined inclusion and exclusion criteria corresponding with the research question(s). We included all studies that met our criteria based on the **P**opulation, **I**ntervention/issue, **C**omparison, **O**utcome, **S**tudy type (PICOS) tool [33] (Table 3).

### 2.4. Data Extraction, Charting, and Synthesis

We extracted the following information about each study: author(s), year of publication, type of study, objective of the study, location of the study, type and method of the study, location and time of the study, population size, age of the population, name of the outbreak/pandemic, mental health issues, instruments used to identify the mental health issues, factors associated with the relevant mental health issue, coping strategies, and occupations of the participants. Microsoft Excel 2016 and Microsoft Word 2016 (Microsoft Corporation, Redmond, Washington, DC, USA) were used to compile and organize the data. All data were compared and contrasted by the authors both individually and conjointly. We undertook descriptive analyses to outline the study literature and to detect the scope of research on the mental health issues of non-health essential workers, including attributable factors and coping strategies. Results were analyzed and discussed to identify key themes and findings.

### 2.5. Interpreting and Reporting Results

The goal of a scoping review is to retrieve a larger general description of the research that has been undertaken on a topic. Through the charting in stage four, we delineated the mental health issues experienced by different groups of essential workers, factors affecting them, and coping strategies. We documented seven groups of non-health essential workers and reported our findings according to the following categories: (1) factory and production occupations; (2) farming, fishing, agriculture, and forestry occupations; (3) food preparation and serving occupations; (4) installation, maintenance, cleaning, and repair workers; (5) sales and related occupations; (6) social care practice and support; and (7) Others.

## 3. Results

### 3.1. Literature Search Overview

**Figure 1:**
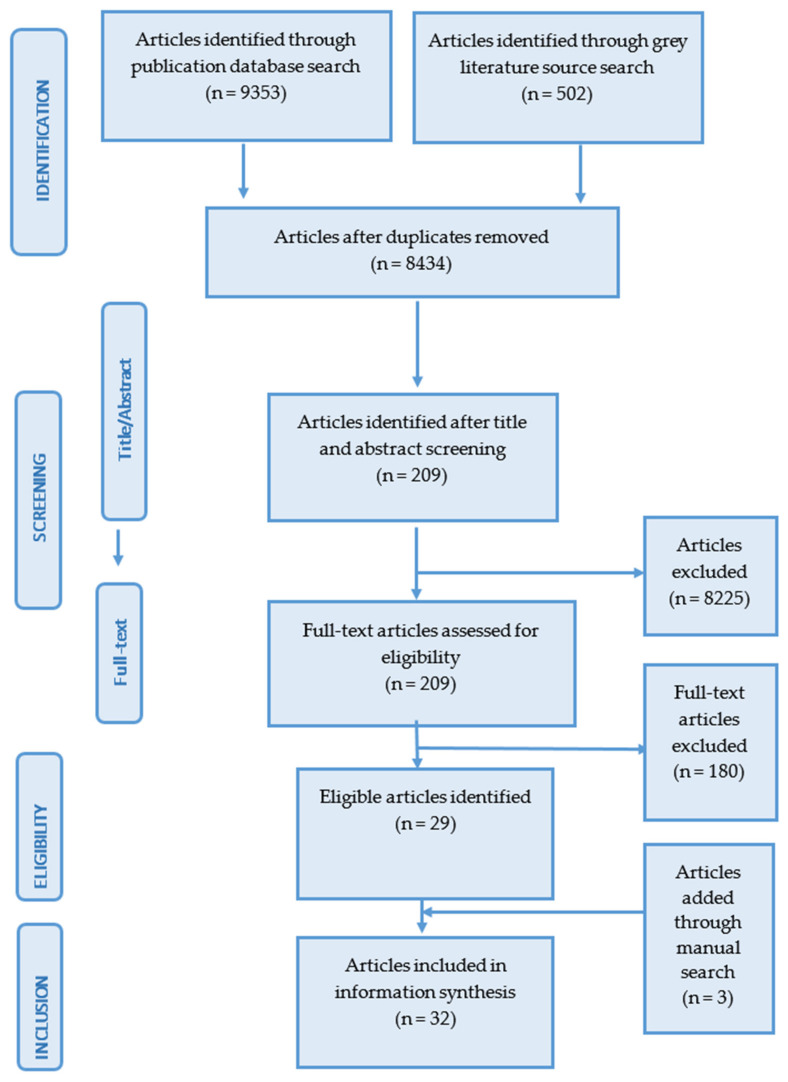
Search and screening steps for this review.

A systematic search of four academic databases yielded 9353 articles. An additional 502 articles were identified through Google Scholar as grey literature. After removing duplicates using Mendeley Reference Manager 2021 software (Mendeley Ltd., London, UK), we undertook the title and abstract screening of 8434 articles. Following screening, 209 articles remained for full-text screening. Full-text screening yielded 29 articles eligible for this review. Through hand searching, we identified three additional articles for a final total of 32 eligible articles (Figure 1).

#### 3.1.1. Content Overview

Table 4 demonstrates the study characteristics extracted from the eligible studies. Of the 32 articles, 20 studies were quantitative, 10 were qualitative, and 2 were mixed methods. Almost all articles pertained to COVID-19, except for one on Ebola. Most of the studies regarding COVID-19 were conducted during March–June 2020 (29 of 32). Most studies were conducted in Spain (n = 9), followed by the USA (n = 7), China (n = 6), and Serbia (2 studies). One study was conducted in each of India, the UK, Turkey, Australia, New Zealand, Bangladesh, and Liberia. Three studies covered several regions that included Brazil, Kenya, Ireland, Canada, Austria, Germany, Switzerland, the Netherlands, and Hong Kong.

#### 3.1.2. Mental Health Issues

Three common mental health issues—anxiety, depression, and stress—were explored by most of the studies. Stress or distress was studied the most (n = 18), followed by anxiety and depression (n = 13, respectively). Other mental health issues included in the studies were worries (n = 2), non-specific mental health issues (n = 2), fear (n = 2), emotional turmoil (n = 2), burnout/workload (n = 2), sleep quality/insomnia (n = 2), alcohol disorder (n = 1), and decreased self-esteem (n = 1).

#### 3.1.3. Instruments Used for Identifying the Mental Health Issues

We also extracted information on what instruments or scales the researchers used to identify the various mental health issues. Twenty-seven studies reported a total of 34 types of validated instruments/scales for diagnosing various types of mental health issues. We organized them into eight groups: anxiety, depression, stress and distress, fear and worries, mood and emotional effects, general mental health and quality of life, sleep and burnout, and others. Ten studies did not report any validated instrument/scale for determining mental health issues. Some of these studies used one-item questions for different mental-health-related issues and/or used questionnaires they developed. While many studies used specific scales for anxiety, depression, stress, or other mental health issues (such as the DASS-21, GAD-7, or MBI scales; see Table 5), some studies used broader general mental-health-related scales (such as Goldberg’s GHQ-12 General Health Questionnaire; see Table 5).

#### 3.1.4. Study Objectives

Most of the studies explored the effect of the pandemic (COVID-19 and Ebola in one study) on the mental health issue of interest. A few studies investigated further, to identify the factors affecting the issues and even fewer to understand the coping mechanisms employed by their target population group. Some studies compared the effects of the pandemic on different professions. Two studies explored how exposure to information, knowledge, and beliefs affected individuals’ mental health.

#### 3.1.5. Data Collection Strategies

Most of the studies used surveys (n = 24) to collect data from individuals. Among them, 10 surveys were conducted using online questionnaires. Seven studies used interviews as their method of data collection, one of which was conducted online. One study used roundtable discussions and in one study the author reflected on their experience using that format. Only two studies used mixed methods that collected data through a survey, focus groups, interviews, or free responses through a survey. The focus group was conducted over Zoom and the interviews were conducted over the telephone.

#### 3.1.6. Outbreaks

COVID-19 was studied in all the reviewed articles except for one on Ebola. We found no studies that explored the mental health effects on non-health essential workers caused by the other four large outbreaks in the last 20 years (2000–2020)—SARS, H1N1 influenza, Zika virus, and MERS.

### 3.2. Mental Health Effects of Outbreaks by Non-Health Essential Occupations

#### 3.2.1. Factory and Production Occupations

Individuals involved in any type of industrial production and manufacturing were included in this category (Table 6). Examples included factory workers, electronic device manufacturing, beverage manufacturing, biopharmaceutical-related industry workers, and more. One study that compared factory and production workers during the pandemic with the pre-pandemic general population found an increased prevalence of depression among factory and production workers (5.6% vs. 2.4%) [45]. Sleep quality among these workers was good to excellent for most (69.5%). Another study found female participants had a higher risk of depression than their male counterparts (n = 66 vs. n = 30) [46].

#### 3.2.2. Farming, Fishing, Agriculture, and Forestry Occupations

A study found that the farmers/workers experienced comparatively less stress (11.5%) than that of the health workers (12.5–16.7%) [35]. A study that investigated farmworker families (where either the woman or her spouse was a farmer) found women in farmworker families experienced fear, worry, and anxiety about being sick and unable to look after their children. They were afraid they might contract COVID-19 from their workplace, and some stopped working to care for their children at home since schools were closed and they could not afford childcare [38].

#### 3.2.3. Food Preparation and Serving Occupations

Individuals in the service sector, including food service and delivery workers, were found to feel less stress (4.4% vs. 6.3%) and anxiety (13.3% vs. 23.0%) compared to those in the health sector according to the DASS-21 scale [40]. Experts in a roundtable discussion expressed concern over the increased anxiety among low-wage essential workers, including food and dairy workers due to their uncertainty about the future and employment [43]. The fear of contracting COVID-19 and infecting others, especially their loved ones, placed them under constant stress. It was also mentioned that many of these populations were immigrants and had joint families, including older parents, at home. In addition, despite there being a substantial number of deaths among this population due to COVID-19, it was unknown if they were provided with any resources to grieve the demise of their co-workers [43].

A study that included health and non-health workers from food and other non-health essential services as participants, but which did not distinguish between them, found a high prevalence of depression, anxiety, and comorbidity amongst the groups (8.3%, 11.6%, and 27.4%, respectively) [10]. Another study reported that 10 of 27 participants in their study (37%) experienced post-traumatic stress disorder (PTSD) symptoms, with women being the most likely to report symptoms (eight out of twenty). Another study categorized the mechanism of COVID-19 affecting individuals in the service sector into four themes: (1) being infected and infecting others during work or transportation to and from work; (2) uncertainties regarding the virus, including its effects on their health and jobs; (3) feeling isolated because they were not able to meet with other people; and (4) work and customer demands, including mixed messages from employers, understaffing, lack of protective resources, and customers not adhering to protective measures (such as mask-wearing) [23].

#### 3.2.4. Installation, Maintenance, Cleaning, and Repair Workers

A study that included health and non-health workers from cleaning and other non-health essential services as participants, but which did not distinguish between them, found a high prevalence of depression, anxiety, and comorbidity amongst these groups (8.3%, 11.6%, and 27.4%, respectively) [10]. They compared Brazil and Spain and found that all symptoms were significantly higher in Brazil than in Spain [10]. A study exploring the anxiety of domestic workers found that 25% of their sample had probable anxiety during the pandemic [13]. One study reported that ageing workers in housekeeping jobs were worried about working in COVID-19 units. They experienced increased stress levels when they realized individuals on the units were more vulnerable to COVID-19 because of their advanced age and comorbidities [43]. Researchers in one study expressed concerns that some less regulated groups of essential workers, such as housekeepers and landscape workers, were not covered by COVID-19 prevention plans that subsequently put them at greater risk [43].

#### 3.2.5. Protective Service Occupations

Anxiety (13.9%) and depression (36.4%) were prevalent among community workstation staff-policemen-volunteers [42]. Protective service workers were reported to be less likely to have a severe psychological impact (26.5%) compared to other essential workers, including healthcare workers (73.6%), grocery workers (65.2%), and media professionals (48.6%) in one study [8]. Another study found protective service workers were impacted psychologically similarly to the general population. However, they were worried about passing the infection on to others, and making them follow COVID-19 regulations such as staying home added to their stress [12]. A study that included armed forces and specialized state security forces found that while 26.4% of the participants felt a current need for psychological help, 52.6% felt they would probably need psychological help if a new wave of the pandemic were to arise. Using the Maslach Burnout Inventory (MBI) subscale, they showed 28.9% had experienced burnout [47]. A study on military personnel found low anxiety and depression levels among the participants, but the participants (65.9%) expressed concerns that despite protective measures they could still contract COVID-19 [50].

#### 3.2.6. Sales and Related Occupations

A study involving retail grocery store workers found that 24% of participants had at least mild anxiety. Workers with anxiety were less likely to report being able to follow COVID-19 regulations at work consistently than those without anxiety (e.g., social distancing, 46% vs. 76%; masking, 63% vs. 84%). A similar finding was observed in workers with depression [7]. Another study found that 65.2% of grocery workers had experienced severe psychological impact due to COVID-19. Over half of the grocery worker participants (56.2%) also mentioned they were depressed. Female grocery workers were found to have higher psychological impacts than males [8]. Another study on fashion retail workers found that COVID-19 caused an increase in their mental workload, which was associated with burnout syndrome among them. Women had higher levels of emotional exhaustion, a burnout dimension, higher levels of emotional demands, and a mental workload dimension, while men showed a higher level of cognitive demands and performance requirements, which are mental workload dimensions [55].

#### 3.2.7. Social Care Practice and Support

A study reported that 46.4% of child welfare workers experienced mild or severe distress. Being single, experiencing financial hardship, having poor physical and mental health, young age, and being non-heterosexual were associated with a higher level of COVID-19 peritraumatic distress [34]. A qualitative study that interviewed family caregivers revealed there were seven types of concerns that caregivers encountered: social isolation of a family member and caregiver, a decline in the mental health of a family member, a decline in the physical and cognitive functioning of a family member, keeping family members safe from COVID-19, lack of caregiving support, and caregiver stress [41]. According to a study that was conducted in the Jiangsu province of China, the top three sources of stress among community workers were being worried about passing on the infection to family members from work (75.5%), personally contracting COVID-19 (71.2%), and non-cooperating residents (67.2%). However, they had better self-rated mental health compared to residents in other areas of China [52]. Another study exploring informal caregivers found that those who provided care to their family members reported lower mental health dimension scores compared to those providing care for non-family members [53].

#### 3.2.8. Transportation and Delivery Occupations

A study that interviewed rickshaw pullers (individuals who pull a three-wheeled passenger cart) who had lost their income amid the pandemic found they experienced psychological stress as a result. Most of the respondents in the study reported being anxious over uncertainty regarding buying food, paying rent, and supporting their family [49]. A study that included transport workers in their non-health essential worker participants found a higher level of depression, anxiety, and stress among them compared to healthcare workers [10].

#### 3.2.9. Other

One study that included media professionals as non-health essential workers found that about half of them had a severe psychological impact (48.6%). About 37% of respondents also expressed being depressed [8]. We found three studies that were conducted among non-health essential workers that did not mention any jobs specifically. They reported 65.1% of respondents had psychological distress that differed by gender (females, 71.6%; males, 52.4%) [36,37,44].

### 3.3. Factors

#### 3.3.1. Demographic Factors

##### Age

Some studies reported that a younger age affected the mental health of non-health essential workers [8,10,45], while one reported that older people may have added stress due to their advanced age and comorbidity being risk factors for COVID-19 and its complications [43].

##### Sex, Gender, and Sexual Orientation

Some studies reported gender and some reported sex. However, in both of these studies, women and/or females showed higher levels of psychological impacts [8,10], including anxiety [50], depression [45], stress [52], and burnout [55]. Some studies did not find any differences [59]. Heterosexual or straight individuals were less likely to have psychological effects than non-heterosexuals [34].

##### Marital Status

One study found that marital status had no impact on the psychological effects of COVID-19 [8]. Another study found married individuals had a lower distress score than singles [34].

##### Education Level

Education level was found to be a protective factor against psychological impacts such as stress [8]. Essential workers with a higher level of education showed less depressive symptoms and better sleep quality [45].

##### Regions

One study found that people living in regions more severely impacted by COVID-19 reported higher psychological impacts [8]. In contrast, another study that compared Brazil and Spain found depression, anxiety, and comorbidity of both were higher in Brazil than Spain, despite Spain having four times the number of COVID-19-related deaths than Brazil during the time they collected their data [10].

##### Financial Status and Expenses

A study reported the monthly income of essential workers, including factory and manufacturing workers, was associated with their depressive symptoms and sleep quality [45]. Another study found that individuals who always have extra money had lower distress scores than those who could not or could barely make ends meet [34]. Increased expenses due to the pandemic were also found to be associated with higher levels of depression, anxiety, and stress [40].

#### 3.3.2. Work-Related Factors

##### Risk of Exposure

Participants in most of the studies reported that the risk of being infected and subsequently infecting one’s family members and loved ones was a source of their worries and anxiety. Advanced age housekeepers indicated concerns about being assigned to COVID-19 units and the high risk of exposure [43]. Domestic helpers showed higher levels of anxiety if they were working in a crowded household [13]. Grocery retail workers who were less likely to practice social distancing or commuted using public transport/ridesharing were more likely to be depressed [8].

##### Increased Work Hours and Workload

Overworking and increased workload due to the pandemic was related to increased psychological impacts for grocery workers and media professionals [8]. Domestic workers also reported an increased workload as their source of anxiety [13]. Community work administrators also felt stressed due to increased work hours [52].

##### Being Unable to Care for Children and See Families

A study investigating farmworker households revealed that the women in those households were afraid they would contract COVID-19 at work and would be unable to take care of their children. Moreover, many had to reduce their hours or leave their job to look after their children because they could not afford childcare costs (as schools were closed) with their low-paying jobs [38]. Police personnel in one study expressed their concerns about being unable to spend time with their family during the lockdown [12].

##### Lack of Workplace Support

One study reported that individuals felt stressed over not having daily allowances of food and refreshments given that restaurants were likely to be closed during the lockdown [12]. A lack of available protective equipment was also expressed as a source of stress and anxiety [12]. Domestic helpers were also worried about getting fired if they contracted COVID-19 [13]. Community work administrators also expressed that not wearing disposable protective equipment caused them stress [52].

#### 3.3.3. Other Factors

According to one study, face-to-face communication and exposure to web-based information about COVID-19 might be associated with increased depressive symptoms and worsened sleep quality. However, positive information regarding COVID-19 such as effective treatments, vaccines, etc., was associated with better mental health [45]. Having had a previous diagnosis or treatment for mental health disorders in the past year and low self-rated health were also associated with higher levels of depression, anxiety, or both [10]. Worrying about the fate of the pandemic, an uncertain future, and physical well-being were also related to higher mental workload and burnout [55].

### 3.4. Coping Strategies

#### 3.4.1. Individual-Initiated Coping Strategies

Some individuals found that applying active coping strategies (constructive behaviour, actively acquiring and applying knowledge, etc.) was useful in coping with stress [35]. Spending time outdoors and daily exercise were reported to be useful coping strategies [23]. Others reported watching movies, listening to music, and reading books helped them to cope with their mental health condition. Some found spending time with family and engaging in hobbies useful. Avoiding news about COVID-19 and thinking about the situation were used as coping strategies by some. While most of these reported practices were harmless, one study reported that some participants used potential harmful activities such as drinking alcohol, smoking, and social avoidance as their way of coping [23].

#### 3.4.2. Available Support

Individuals expressed that they may need psychosocial support to cope with the effects of the pandemic. The availability of protective equipment and a spirit of teamwork cultivated in the workplace were also helpful [47]. Receiving support from employers was reported as being helpful by some professionals such as grocery workers in one study [8]. This study also reported that those grocery store workers who received economic support during the pandemic found it helpful for reducing the psychological impacts of the pandemic [8].

## 4. Discussion

The objective of our study was to identify the effects of the six pandemics that have occurred in the last 20 years (SARS, H1N1 influenza, Ebola, Zika virus, MERS, and COVID-19) on non-health essential workers. We found that, other than one article on Ebola, all articles involved COVID-19. None of the other five pandemics lasted as long as COVID-19 has, did not result in as much loss of life, or did not uproot the existing societal infrastructure as greatly. For some reason, researchers did not consider exploring the mental health of non-health essential workers during those other pandemics. The articles we found discussed the mental health implications of COVID-19 on seven types of non-health essential jobs, which we categorized as (1) factory and production occupations; (2) farming, fishing, agriculture, and forestry occupations; (3) food preparation and serving occupations; (4) installation, maintenance, cleaning, and repair workers; (5) sales and related occupations; (6) social care practice and support; and (7) Others. The studies reported many mental health issues such as anxiety, depression, stress and distress, fear and worries, mood and emotional effects, general mental health and quality of life, sleep and burnout, and others. Most of the studies found evidence of an increase in these mental health effects when compared with the general population and/or health professionals. These increased effects may have arisen from a lack of adequate training against protection from the disease, a lack of supply of protective equipment, and a lack of sufficient support from employers.

One major concern of non-health essential workers from across sectors was a worry about infecting others, especially older parents and children contracting COVID-19. This concern was also prevalent among healthcare workers as found in other studies [60]. Non-health essential workers were also overworked, which led to burnout and additional stress. Similar reports were documented regarding healthcare workers [61]. Unlike healthcare workers who were praised everywhere and considered “superheroes”, we did not find any studies reporting a similar phenomenon amongst non-health essential workers. On the contrary, some workers mentioned it had been a rather unpleasant experience and that they felt stigmatized by friends and family who avoided and kept their distance from them [48]. A lack of supply of PPE was mentioned by non-health essential workers as being stressful in several studies. Early during the pandemic the media highlighted the lack of adequate protective equipment for health workers [62], which helped raise awareness of the issue. However, despite encountering similar difficulties and being at comparable risk of contracting COVID-19, the concerns of non-health essential workers were not considered equally.

One of the factors affecting the mental health of non-health essential workers was inadequate training to protect themselves against COVID-19. This factor was less likely a concern for healthcare workers, since in addition to regular COVID-19-specific training at their workplace [63], they had the foundational knowledge to address how this type of virus was transmitted and what protective measures to take to prevent it from spreading. Mixed messages from employers and exposure to media, social media, and others also contributed to increasing their stress and other mental health effects, which were found to affect the general population [28] and healthcare workers similarly [64]. Generally, women were reported to be more vulnerable to experiencing mental health effects due to the pandemic than men, except among those who worked as protective service workers or media professionals [8]. One study pointed out that this phenomenon could be related to the overall higher rate of mental health disorders among women, which may be linked to a different neurobiological response in women compared to men and the overrepresentation of women who work in grocery, retail, the service industry, and healthcare [28,65]. A younger age, lower education level, being unmarried, and being migrant workers also arose as affecting the mental health of non-health essential workers in our sample of articles, which were also found in a systematic review of the effects of COVID-19 on the general population [28]. Interestingly, one study found that people in Brazil were more affected by mental health effects due to COVID-19 compared to those in Spain, despite the number of deaths due to COVID-19 being four times higher in Spain than Brazil. The researchers reasoned that additional social, structural, and political problems in Brazil may have exacerbated the mental health effects of the pandemic [10].

Coping strategies were predominantly initiated by non-health essential workers themselves and included physical activities, positive thinking, relaxation, and communicating with friends and family. These activities have been supported by previous studies on mental health as being good practice [66,67]. Nevertheless, some self-harming coping mechanisms such as increased smoking, consuming alcohol, and suppressing emotions also arose. Similar practices, particularly increased drinking, have also been found in healthcare workers [68] and the general population [69]. Substance use is another negative coping mechanism people often practice [70]. We did not find any evidence of substance use in our reviewed articles; however, the studies did not specifically explore that issue and people might be more conservative about sharing this type of information [71]. In some studies, participants mentioned having institutional support from their workplace [7], whereas others mentioned a lack of support [39]. However, no detailed information on the type and extent of the supports was explored.

A strength of our study was that we undertook a comprehensive search to capture any research on mental health effects of pandemics on non-health essential workers. The research questions were clear, and we were able to identify and synthesise relevant findings from the studies in our sample. However, we only found studies on COVID-19 and Ebola on this topic and population. Despite our best efforts, this review also has some limitations. We tried to identify findings for different occupations within non-health essential workers; however, only a few studies involved occupation-specific roles, rather presenting their findings as part of a larger population group. The effects of a pandemic may not be the same for each of the non-health essential worker groups, but we could not address that in this review. We included articles from all sources, including qualitative and descriptive quantitative studies (all of which were cross-sectional), but we could not establish a causal relationship between mental health issues and the factors affecting non-health essential workers. Further, while we included studies from diverse methodologies in our review, we did not assess the quality of the literature. In addition, only a handful of studies addressed the coping strategies of participants, which did not provide us with a comprehensive understanding of this important aspect of mental health.

## 5. Conclusions

This scoping review compiled knowledge about the effects of recent pandemics on the mental health of non-health essential workers, who play a crucial role during a pandemic. A lack of non-health essential occupation-specific research on this topic warrants a future detailed study on this population group. Some qualitative and cross-sectional studies explored various factors related to mental health during pandemics; however, more longitudinal studies would help establish any causal inferences. Coping strategies is another important area for future research. Moreover, studying the post-pandemic mental health effects of COVID-19 would be useful to provide a baseline for comparison purposes. The efforts of non-health essential workers were not sufficiently recognized during this pandemic. Based on our learnings from this study, we surmise that these individuals might not receive the support required to help them recover from their mental health issues. Developing mental health support and rehabilitation programs and strategies specific to these various non-health essential workers is highly recommended. Furthermore, the knowledge gained through this study can be used to help design public health measures to help mitigate the effects of the pandemic on other groups critical to the social and economic welfare of populations and help them recover quickly from the socioeconomic effects of the pandemic.

## Figures and Tables

**Table 1:** Search terms and strategies.

A. Mental-health- and wellness-related terms Mental health [MeSH, Keyword] OR Mental wellness [Keyword] OR mental wellbeing [Keyword] OR psychological wellbeing [Keyword] OR anxiety [MeSH, Keyword] OR anxiety disorders [MeSH, Keyword] OR anxiety symptom* [Keyword] OR generalized anxiety [Keyword] OR social anxiety [Keyword] OR psychological stress [Keyword] OR Stress, Psychological [MeSH] OR Occupational Stress [MeSH, Keyword] OR post-traumatic stress [Keyword] OR Stress Disorders, Post-Traumatic [MeSH] OR cognitive function* OR Cognition Disorders [MeSH, Keyword] OR Cognitive Dysfunction [MeSH, Keyword] OR Mental Disorder* [MeSH, Keyword] OR Mood disorder* [MeSH, Keyword] OR depress* mood [Keyword] OR Bipolar Disorder* [MeSH, Keyword] OR depress* symptom* [Keyword] OR Depressive disorder, Major [MeSH] OR Depressive Disorder [MeSH] OR depressive disorder* [Keyword] OR dysthymia [Keyword] OR Dysthymic Disorder [MeSH] OR manic depression [Keyword] OR Obsessive–Compulsive Disorder [MeSH] OR Phobic Disorder* [Keyword, MeSH] OR brief psychotic [Keyword] OR Psychotic Disorder [MeSH] OR schizoaffective disorder* [Keyword] OR paranoid [Keyword] OR Paranoid Disorders [MeSH] OR antisocial [Keyword] OR Antisocial Personality Disorder [MeSH, Keyword] OR Substance-Related Disorders [Keyword, MeSH]
B. Disease-outbreak-related terms infectious disease [keyword] OR Communicable disease [MeSH] OR virus OR viruses [MeSH] OR outbreak [keyword] OR Disease Outbreaks [MeSH] OR Ebola OR Ebola Vaccines [MeSH] OR Hemorrhagic Fever, Ebola [MeSH] OR Zika OR Zika Virus [MeSH] OR Zika Virus Infection [MeSH] OR SARS [Keyword] OR SARS virus [MeSH] Coronavirus Infections [MeSH] OR Betacoronavirus [MeSH] OR Coronavirus [Keyword, MeSH] OR Severe Acute Respiratory Syndrome [MeSH] OR MERS [keyword] OR Middle East Respiratory Syndrome Coronavirus [MeSH] OR Swine flu [Keyword] OR Influenza A virus, H1N1 Subtype [MeSH] OR COVID-19
C. Worker/ethnic-minority-related terms factory worker [Keyword] OR warehouse worker [Keyword] OR worker [Keyword] OR retail worker [Keyword] OR industrial worker [Keyword] OR Community Health Worker [MeSH] OR Health Personnel [MeSH] Searched using (All ‘A’ terms) AND (All ‘B’ terms) AND (All ‘C’ terms)

**Table 2:** Academic and grey literature databases used to search eligible studies.

Academic Databases	Grey Databases
MEDLINE PsycInfo CINAHL Sociological Abstracts Web of Science	Google Scholar

**Table 3:** Inclusion and exclusion criteria.

	Inclusion Criteria	Exclusion Criteria
Population	Essential workers	Health-related essential workers
Intervention/issue	Any mental-health-related issues and intervention, including depression, anxiety, stress, sleep quality, etc.	Not applicable
Comparison	Not applicable	Not applicable
Outcome	Participant-reported mental health issues, factors associated with those issues, and coping strategies adopted by the participants or supports provided	Any outcome not originating from mental health issues due to COVID-19, such as mental health issues related to certain stressful jobs regardless of COVID-19 (e.g., police and firefighters)
Study type	Primary research including observational and experimental studies, qualitative studies, field report, and case studies	Not applicable

**Table 4:** Description of study characteristics.

Study	Study Objective	Study Type	Data Collection	Time of the Study	Location of the Study	Population Size (n)	Age (y as Mean or Range)	Study Population	Outbreak Name	Mental Health Issue
Miller et al. (2020) [34]	To explore whether COVID-19 caused the child welfare worker any peritraumatic distress	Quantitative	Online survey	Summer of 2020	USA	1996	41.44	Male 188, female 1804, other 4; 800 employed by a private child welfare agency; 1196 employed by a public child welfare agency	COVID-19	Peritraumatic distress; overall child welfare workers fall under the mild distress category
Du et al. (2020) [35]	To evaluate the burden of mental health issues on several professions in China to identify vulnerable groups, factors influencing the issue, and better coping mechanisms	Quantitative	Online survey	March to April 2020	China	687	36.92	158 doctors, 221 nurses, 24 other medical staff, 43 students, 60 teachers/government staff, 135 economy staff, 26 workers/farmers, and 20 professions designated under the “other” category	COVID-19	Depression, anxiety, and stress
Ruiz-Frutos et al. (2021) [36]	To study the differences between the mental health of non-health workers who work from home and those who work away from home (essential non-health workers)	Quantitative	Survey	March to April 2020	Spain	1089 workers; 494 away from home, 597 from home	42.1	Currently active worker; adult	COVID-19	Psychological distress
Ruiz-Frutos et al. (2021) [37]	To assess the effects of COVID-19 on the physical and mental health of non-health workers	Quantitative	Survey	March to April 2020	Spain	1089 workers; 494 away from home, 597 from home	42.1	Currently active worker; adult	COVID-19	Psychological distress
Boovaragasamy et al. (2021) [12]	To explore the perception of police personnel towards COVID-19 and the factors influencing their stress and coping abilities amid COVID-19	Qualitative	Interviews	April of 2020	India	32	25–60	Police personnel; 78.12% were married, 62.5% were in the profession for over 5 years	COVID-19	Stress
Quandt et al. (2021) [38]	To explore the experience of women in families during COVID-19 in work and household economics, childcare and education, health care, and the community social climate concerning discrimination and racism	Quantitative	Survey	May to June 2020	USA	105	25–47	Female Latinx farmworkers and female non-farmworkers	COVID-19	Stress, worry about contracting COVID-19, worry about children
De Camargo (2021) [39]	To explore the fears and anxiety of contracting COVID-19 in police officers	Qualitative	Online interviews	May to June 2020	United Kingdom	18	22–54	88.8% were married or in a relationship, average experience of 10 years	COVID-19	Stress, the safety of family and others
Kabasakal et al. (2021) [40]	To evaluate the depression, anxiety, and stress status of health sector and community service workers who were actively working during the pandemic	Quantitative	Survey	May to June 2020	Turkey	735	45 years and older	Health and service sector employees	COVID-19	Depression, anxiety, and stress
Lightfoot et al. (2021) [41]	To explore the concerns and benefits of family caregivers during the COVID-19 pandemic	Qualitative	Interviews	May and September 2020	Minnesota, USA	52	58	Family caregivers	COVID-19	Concerns about the mental and physical health of elderly family members, keeping them safe from COVID-19
Li et al. (2021) [42]	To determine the prevalence of anxiety and depression related to the COVID-19 pandemic in quarantined people, community workstation staff-policemen-volunteers (CPV) and the general public, and to examine their potential risk factors	Quantitative	Survey	March 2020	Hubei, China	3303	18 years and above	General medical staff, the general public, front-line health care workers, community workstation staff-policemen-volunteers (CPV) and quarantined people	COVID-19	Anxiety, depression
Gallagher et al. (2021) [43]	To explore the concerns and needs of low-wage essential workers as understood by experts in the field	Qualitative	Roundtable discussion	N/R	USA			Risk manager/analysts, a professional health educator/trainer, senior leaders, and occupational health professionals were invited to participate; representatives from the dairy, childcare, food bank, and healthcare industries were included	COVID-19	Non-specific mental health
Lan et al. (2021) [7]	To investigate SARS-CoV-2 infection and exposure risks among grocery retail workers, and to investigate their mental health state during the pandemic	Quantitative	Survey	May 2020	Massachusetts, USA	104	18 and above	Grocery retail store employees	COVID-19	Anxiety and depression
Toh et al. (2021) [29]	To characterize the concerns endorsed by health care workers and other essential workers relative to the general population and to explore differences among these groups	Quantitative	Survey	April 2020 to March 2021	Australia	5158	18 and above	Essential workers, non-essential workers, and the general population	COVID-19	Depression, anxiety, and stress
Ruiz-Frutos et al. (2020) [44]	To explore what influenced the level of psychological distress in a sample of non-health workers in Spain	Qualitative	Survey	March to April 2020	Spain	1089	18 and above	Non-health workers	COVID-19	Psychological distress
Pan et al. (2020) [45]	To investigate the associations between COVID-19-specific information exposure and four outcome variables, including depression, sleep quality, self-reported consistent face-mask-wearing, and hand hygiene	Qualitative	Survey	March 2020	Shenzhen, China	3035	18 and above	Factory workers	COVID-19	Depression, sleep quality
De Boni et al. (2020) [10]	To assess the prevalence and predictors of depression, anxiety, and their comorbidity among essential workers in Brazil and Spain during the COVID-19 pandemic	Quantitative	Survey	April to May 2020	Brazil and Spain	3745	Adults	Essential workers	COVID-19	Depression, anxiety
Fang et al. (2020) [46]	To explore mental health outcomes in non-medical volunteers who worked in Wuhan fighting COVID-19, especially to understand the psychological status of volunteers born after the 1990s	Quantitative	Survey	February to March 2020	Wuhan, China	191	>20 years	Non-medical workers	COVID-19	Positive and negative affect, perception of stress, depression, and emotional state
Yeung et al. (2020) [13]	To examine the psychosocial correlates of probable anxiety among Filipina domestic helpers in Hong Kong amid the COVID-19 pandemic	Quantitative	Survey	May 2020	Hong Kong	295		Filipina domestic workers	COVID-19	Anxiety, COVID-19- specific worries, social support
Rodriguez-Rey et al. (2020) [8]	To explore the psychological symptomatic response of frontline workers working during the COVID-19 pandemic and the potential demographic and work-related factors that may be associated with their symptoms	Quantitative	Online survey	March to June 2020	Spain	546		Healthcare workers	COVID-19	Psychological impact, depression, degree of concern
Lazaro-Perez et al. (2020) [47]	To understand the level of anxiety in the face of death in essential professionals and to determine the predictive variables involved in this phenomenon	Quantitative	Survey	August to September 2020	Spain	2079	Up to 30 years old, 31–40 years old, 41–50 years old, 51–60 years old, and over 60 years old	Military personnel of the Armed Forces, National Police and Civil Guards	COVID-19	Death, anxiety, fear of one’s death, fear of the process of dying, fear of death of others, fear of the process of others dying
Zolnikov et al. (2020) [48]	To understand stigma experienced by first responders during the COVID-19 pandemic and the consequences of stigma on first responders’ mental health	Qualitative	Interviews	N/R	USA, Kenya, Ireland, Canada	31	above 18	Healthcare workers and first responders	COVID-19	Depression, anxiety, feeling isolated, stress, insomnia, decreased self-esteem
Ahmed and Sifat (2020) [49]	To understand the social, economic, and mental health effects on the lives of deprived and marginalized rickshaw pullers in Bangladesh during the COVID-19 situation	Qualitative	Interviews	N/R	Bangladesh	11	18 or above	Informal workers	COVID-19	Anxiety, stress, depression
Markovic et al. (2020) [50]	To assess the prevalence and degree of anxiety and depression in education, army, and healthcare professionals	Quantitative	Online survey	July 2020	Serbia	110	Not specified	Skilled workers	COVID-19	Depression, anxiety
Teng et al. (2020) [51]	To explore COVID-19-related depression, anxiety, and stress among quarantined hotel employees in China	Quantitative	Online survey	May to June 2020	China	170	Not specified	Quarantined hotel employees	COVID-19	Depression, anxiety, and stress
Zhang et al. (2020) [52]	To describe the physical and mental health of community workers and explore the associated factors	Quantitative	Online survey	February to March 2020	China	702	18 years or older	Community workers	COVID-19	Stress, emotional effects, and mental health
Rosemberg et al. (2021) [23]	To explore the perspective of workers regarding the effect of COVID-19 on their mental health and coping, including screening for post-traumatic stress disorder (PTSD) and alcohol use disorder symptoms	Qualitative	Interviews	N/R	USA	27	18 or older (mean 37 years)	Adult, English-speaking, food retail, food service, or hospitality industry workers, residing in one of the selected 10 states based on COVID-19 case counts per 100,000 population	COVID-19	Post-traumatic stress disorder (PTSD) and alcohol use disorder symptoms
Todorovic et al. (2020) [53]	To assess the quality of life of informal caregivers during the COVID-19 epidemic in Serbia	Mixed	Survey, focus groups (via Zoom), interviews (telephone)	March to May 2020	Serbia	112	51.1 ± 12.3	Informal caregivers	COVID-19	Non-specific mental health
Blanco-Donoso et al. (2021) [54]	To analyze the psychological consequences of the COVID-19 pandemic on nursing home workers and the influence of certain related stressors and job resources to support the stress	Quantitative	Online survey	N/R	Spain	228	36.29	Spanish nursing home workers	COVID-19	Traumatic stress
Rodriguez-Lopez et al. (2021) [55]	To analyze the levels of mental workload and the presence of burnout on a sample of fashion retail workers from Spain and its relationship to the COVID-19 pandemic	Quantitative	Online survey	October and November 2020	Spain	360	32.48	Spanish fashion retail workers	COVID-19	Mental workload and burnout
Cheung (2015) [56]	To summarize the experience and lessons learned from the Ebola virus disease outbreak in Liberia	Qualitative	Field report	N/R	Liberia	NA	NA	NA	Ebola	Fear, rumours, and stigma
Frenkel et al. (2020) [57]	To determine whether facing the COVID-19 pandemic affects police officers as they are confronted with various novel challenges	Mixed	Online survey with free response feature	March to June 2020	Global (Austria, Germany, Switzerland, the Netherlands, and Spain)	2567	39.69	European police officers	COVID-19	Strain
Bell et al. (2021) [58]	To compare psychological outcomes, experiences, and sources of stress over the COVID-19 lockdown in New Zealand in essential workers (healthcare and “other” essential workers) with that of workers in non-essential work roles	Quantitative	Online survey	April to May 2020	New Zealand	2495	18 or over	Healthcare and other essential workers	COVID-19	Anxiety, distress, well-being

**Table 5:** Scales/instruments used to diagnose various mental-health-related issues among participants.

Mental Health Issues	Scales/Instruments Used
Anxiety	Depression, Anxiety and Stress Scale (DASS-21) Generalized Anxiety Disorder-7 (GAD-7) Chinese version of the Zung’s Self-rating Anxiety Scale (SAS) Child Pain Anxiety Symptoms Scale (CPASS) Beck Anxiety Inventory (BAI)
Depression	Chinese version of the Self-rating Depression Scale (SDS) Patient Health Questionnaire-9 (PHQ-9) Patient Health Questionnaire-2 (PHQ-2)
Stress and distress	Depression, Anxiety and Stress Scale (DASS-21) COVID-19 Peritraumatic Distress Index (CPDI) Simplified Chinese version of the 14-item Perceived Stress Scale Stress Reaction Questionnaire (SRQ) Impact of Event Scale-Revised (IES-R) Stress appraisal measure (SAM) Primary Care PTSD Screen (PC-PTSD-5) Social Pressure subscale of the Secondary Traumatic Stress Questionnaire Kessler Psychological Distress Scale (K10)
Fear and worries	Fear of COVID-19 Scale (FCV-19S) Collett–Lester Fear of Death Scale Contact with Death and Suffering subscale of the Nursing Burnout Scale
Mood and emotional effects	Positive and negative mood questions Positive and Negative Affect Schedule (PANAS) German Multidimensional Mood Questionnaire 6-item
General mental health and quality of life	Goldberg’s GHQ-12 General Health Questionnaire European Health Interview Surveys—Quality of Life (EUROHIS-QoL) Short Multidimensional Inventory Lifestyle Evaluation—Confinement (SMILE-C) Short Form Health Survey (SF-36) Short Form Health Survey (SF-12) WHO Well-Being Index (WHO-5)
Sleep and burnout	Sleep Quality Scale Maslach Burnout Inventory (MBI) Workload subscale of the Secondary Traumatic Stress Questionnaire CarMen-Q questionnaire (workload)
Others	Ultrecht Work Engagement (UWES-9) short scale (for engagement) Antonovsky’s sense of coherence 13-item questionnaire (SOC-13) (for coherence) Social Support at Work subscale of the Job Content Questionnaire

**Table 6:** Mental health issues, factors, and coping strategies among different categories of non-health essential workers.

Categories	Occupations (Non-Health Essential Workers)	Study	Main Findings per Study	Factors per Study	Coping Mechanisms
Factory and production occupations	Factory workers	Pan et al. (2020) [45]	Increased prevalence of depression (5.6%) was found The prevalence of good or excellent sleep quality was found (69.5%)	Higher information exposure via unofficial web-based media and face-to-face communication was associated with higher depressive symptoms Age, marital status, education level, monthly personal income, and factory type were significantly associated with depressive symptoms and sleep quality	N/R
	Electronic device manufacturing				
	Watchmaking				
	Beverage manufacturing				
	Biotechnology product manufacturing				
	Biopharmaceutical-related industry workers	Fang et al. (2020) [46]	Depressed participants had a higher perception of stress (emotional reaction, physical reaction, and behaviour reaction) than non-depressed participants	Female participants had a higher risk of depression compared to male participants	N/R
Farming, fishing, agriculture, and forestry occupations	Farmers	Fang et al. (2020) [46]	Approximately 50.3% of frontline non-medical workers, including farmers, were reported to have clinically significant symptoms of severe depression	Post-1990s respondents (the generation born after the 1990s) were more likely to experience depression and have increased negative effects	N/R
		Quandt et al. (2021) [38]	Some of the farmworker households were scared that they might contract COVID-19 from work and infect their kids Some had to leave work to take care of their children, which added to their stress	Needed to work close to others, share rides, unable to wear a mask all the time due to heat, and others taking no precautions worried the participants Lack of government income support for being immigrants and undocumented	N/R
		Du et al. (2020) [35]	77.8% and 19.2% of the participants fell in the normal–mild and moderate–extremely severe category of depression, respectively 26.9% fell in the moderate–extremely severe anxiety category 85.5% and 11.5% fell in the normal–mild and moderate–extremely severe category of stress on the DASS-21	Being female, applying emotional coping strategies such as venting emotions, consuming alcohol, etc., increased the probability of belonging to the moderate–extremely severe depression, anxiety, or stress group	Applying active coping strategies (applying constructive behaviour, actively acquiring and applying knowledge, etc.) were found successful Relaxation techniques, psychoeducation, and promoting social contact were useful
Food preparation and serving occupations	Butchers	Kabasakal et al. (2021) [40]	High stress levels were reported among the participants in the service sectors (4.4%) Participants from these sectors also reported having a high level of anxiety (13.3%) and depression (15.5%)	Increased number of work hours Increased expenditures for cleaning and food Reduced appetite, sleep duration, physical activity Reduced use of social media due to work	Physical activities Spending time outdoors Talking or virtually connecting with friends and family Positive outlook
	Bakers				
	Drinking-water dealers				
	Food services				
	Catering sector workers				
	Food and dairy	Gallagher et al. (2021) [43]	Increased anxiety among the non-essential workers, including food, dairy, and meatpacking industry workers	Uncertainty about the future and employment Living in joint families with older parents	No known grief support for those who lost those close to them
	Meatpacking industry				
	Restaurants				
	Hospitality workers	Rosemberg et al. (2021) [23]	Most commonly experienced symptoms included trying hard not to think about/avoid situations that reminded them of COVID-19, being constantly on guard/watchful, being easily startled, and feeling numb or detached from people, activities, or surroundings	Women were more likely to suffer from PTSD Low case count was related to low PTSD symptoms	Some participants reported having increased alcohol consumption to cope with the effect
	Food retail				
	Food	De Boni et al. (2020) [10]	8.3%, 11.6%, and 27.4% presented positive screenings for depression, anxiety, and both, respectively	An unhealthy lifestyle increased the likelihood of both depression and anxiety	N/R
	Hotel employees	Teng et al. (2020) [51]	43.5% of respondents reported having moderate to extremely severe symptoms of depression, whereas 68.2% of participants had higher levels of anxiety symptoms Severe stress symptoms were seen among 8.2% of participants	Lower monthly income was associated with depression, a lower education level (lower than middle school level or junior college), specific age (millennials) was associated with anxiety Gender (female) was associated with stress	Staying in touch with personal support networks Building positive workplaces Opening protective and private communication pathways Constructing a resilience model
Installation, maintenance, cleaning, and repair workers	Cleaning	De Boni et al. (2020) [10]	Depression, anxiety, and both as comorbid conditions were found	People living in Brazil experienced anxiety and depression more commonly than those in Spain despite there being a 4 times higher rate of COVID-19 deaths in Spain	N/R
	Domestic workers/helpers	Yeung et al. (2020) [13]	25% of female domestic helpers reported having probable anxiety	Lack of PPE, increased workload, and worries about being fired if they contracted COVID-19 were significantly associated with probable anxiety Social support from employers, family, friends, and community organizations was not associated with probable anxiety	Social support, COVID-19 information literacy
	Landscape workers	Gallagher et al. (2021) [43]	These workers may have been forgotten in the COVID-19 prevention plan since they are not generally employed by corporate companies	Being the sole providers of the households may interfere with work and home responsibility balance and lead to fatigue and irritability	N/R
	Housekeeping workers				
	Police officers	Frenkel et al. (2020) [57]	Overall, police officers tolerated the pandemic well; however, strains differed largely between individuals and among countries German officers were most strained, followed by Spanish, Austrian, Dutch, and Swiss officers	Stressor appraisal, emotion regulation, preparedness, sex, and working experience significantly predicted strain during the pandemic	Engaging in maladaptive emotion regulation, i.e., rumination and expressive suppression, was associated with higher strain The use of adaptive emotion regulation, i.e., reflection, reappraisal, social sharing, and distraction, reduced officers’ strain during the pandemic
		De Camargo (2021) [39]	Fear of contracting COVID and fear of passing the infection on to family and loved ones	Inadequate initial testing Lack of support from authorities/organizations	N/R
		Boovaragasamy et al. (2021) [12]	Increased feelings of sadness and being at risk for anxiety and depression Burnout, emotional exhaustion, and depersonalization Reported feelings of isolation Decreased or forced removal in immediate social interaction (e.g., within family and friend circles) Reluctance to ask for help or get treatment	Being unable to spend time with family No daily allowance No PPE Lack of support from management Lack of social support generally Being in quarantine Younger age Increased work hours	Support received from workplace
	Firefighters	Toh et al. (2021) [29]	Concerns about the death of loved ones due to COVID-19, health and well-being of self and society Non-health essential workers, including firefighters, reported having higher levels of depression than healthcare workers They were also more anxious and stressed than healthcare workers	Age, sex, state of residence, and lifestyle (i.e., sleep patterns, anticipated time to the lifting of government restrictions) may have possibly influenced observed mental health outcomes	N/R
	Protective service workers	Rodriguez-Rey et al. (2020) [8]	Most of the protective service workers showed minimum psychological impact (54.2%) The protective service workers showed similar levels of psychological impact as the general population	No gender differences were found in this study	Support received from workplace
	Military personnel	Markovic et al. (2020) [50]	A significant difference with regard to the mean BAI score was observed among different occupation groups (*p* < 0.05) between army and healthcare workers	N/R	N/R
	Civil guards	Lazaro-Perez et al. (2020) [47]	82.1% showed fear of the death of others and 78.2% concerning the process of others dying Higher values were obtained for death anxiety (DA) linked to the death and dying processes of others, much higher than if one asked about one’s death and the dying process 53.8% showed higher levels of emotional exhaustion A higher percentage 58.4% of depersonalization was found 87.8% stated that the absence of PPE increased levels of stress and anxiety during the first wave of the pandemic	Lack of PPE increased stress and anxiety level	N/R
	Police officers	Zolnikov et al. (2020) [48]	Apart from worries due to being COVID-19-positive from exposure due to work, the first responder participants, including police officers, felt stigmatized by friends and family that they might be the source of infection and people recoiled from them	N/R	N/R
Sales and related occupations	Grocery retail workers	Lan et al. (2021) [7]	Over 65% of grocery workers showed a severe psychological impact and a higher impact level than the general population	Being a grocery worker and female Direct customer exposure Public transport, shared rides, and being exposed to a confirmed case were correlated positively with depression	Support received from company/workplace
		Rodriguez-Rey et al. (2020) [8]	65.2% showed severe psychological impact derived from working during the COVID-19 crisis The grocery workers showed significantly higher psychological impacts than the general population Most of the workers were worried about “Infecting a beloved one by coronavirus”	Women showed significantly higher psychological impacts than men Occupation (more stress in grocery workers) The feeling of teamwork was high and was associated with lower psychological impacts	N/R
		Rosemberg et al. (2021) [23]	Around 37 participants had PTSD A higher proportion of women (55%) expressed worry about infecting their family members and others as compared to men (17%)	Heightened levels of mental distress were related to the place of work Already existing disparities they faced on a daily basis before the pandemic started Mixed messages and poor communication from their employers were a source of stress	N/R
	Cashiers	Kabasakal et al. (2021) [40]	There was a high likelihood of stress, anxiety, and depression, ranging from around 4–15%	Increased work hours and expenditures for cleaning and food Uncertainty due to pandemic	N/R
	Booth attendants				
	Supermarket workers	Toh et al. (2021) [29]	Non-health essential workers felt vulnerable and at risk of falling ill due to COVID-19	Lack of training Inadequate safety protocols	N/R
	Fashion retailing workers		Emotional demands and emotional exhaustion as a burnout dimension were associated with participants’ perception of COVID-19 Increased workload is associated with greater cognitive, emotional, temporal, and performance demands	Environmental changes, somatic symptoms, insomnia, negative job expectations, and uncertainty also were significant mental workload predictors Women had higher levels of emotional exhaustion and demands Men had higher levels of cognitive demand and performance requirements than women	N/R
Social care practice and support	Child care/welfare workers	Miller et al. (2020) [34]	Childcare providers were in mild to severe distress	Social isolation declines mental health Being unmarried, non-heterosexual, poor financial status affect mental health	Keeping emotional distance from elderly Maintaining own well-being and kindness
	Community workstation staff-policemen-volunteers	Li et al. (2021) [42]	Anxiety (13.9%) and depression (36.4%) in community workstation staff-policemen-volunteers (CVP) were prevalent	Being in quarantine People at younger ages were more likely to have risks of anxiety and depression compared with people older than 60 Having close contact with COVID-19, having family or friend as front-line health worker Spending time learning epidemic information	N/R
	Community/social workers	Zhang et al. (2020) [52]	The top three stresses of participants included worrying about infecting family members after work (75.5%), worrying about being infected (71.2%), and residents not cooperating (67.2%) Community workers had better self-rated mental health compared to the residents of other places in China	Being community work administrators, not wearing PPE, having to seek help, more work hours, and work pressure sources worsened self-rated mental health	N/R
	Informal caregivers	Lightfoot et al. (2021) [41]	Concerns about keeping family members safe	Social isolation of a family member and caregiver Lack of physical exercise Lack of caregiving support Financial uncertainty	N/R
		Todorovic et al. (2020) [53]	Informal caregivers and those providing care to family members had lower physical and mental health dimension scores in contrast to those providing care for non-family members	Significant predictors of mental health included providing care for a family member, complexity of care required, and increased concerns about self-health and the health of the person being cared for	N/R
	Geriatric assistants	Blanco-Donoso et al. (2021) [54]	In nursing homes where COVID-19 was detected the workers had a higher level of secondary traumatic stress Increased workload, secondary traumatic stress, and fear of contagion were found significant	Social pressure from work (e.g., pressure from relatives) Lack of PPE Lack of adequate supervisor support More exposed to the suffering/death of people	N/R
	Nursing home managers				N/R
	Aid workers	Cheung (2015) [56]	Humanitarian aid workers during Ebola were in fear and stress that they and their close ones may contract Ebola	Stigmatization, including shame or disgrace, was imposed on people related to the disease	Attempting mindfulness techniques to cope with stress
	Logistics and cargo services workers	Toh et al. (2021) [29]	Non-health essential workers, including logistics and cargo services workers, fared worse than healthcare workers regarding elevated stress and dissatisfaction with specific life domains	Inadequate training, lack of safety protocols and practices, and uncertainties associated with a risky profession	N/R
	Transportation workers	De Boni et al. (2020) [10]	Essential workers in this study, which included transportation workers, presented anxiety and depressive symptoms	Political, social, and structural stability of the country	N/R
	Rickshaw pullers	Ahmed and Sifat (2020) [49]	Labourers had drastic income losses that put them under severe socioeconomic and psycho-sociodemographic stress A majority of the respondents were very anxious about money and food during the pandemic	Lockdown, loss of income, and lack of cheap street food (due to lockdown) increased their stress and anxiety	N/R
Other	Media professionals	Rodriguez-Rey et al. (2020) [8]	Media personnel followed healthcare workers and grocery workers in sadness scale Media personnel were less concerned about getting infected than grocery/healthcare workers	No gender difference was found for media professionals	N/R
	Non-specific health care essential workers	Ruiz-Frutos et al. (2021) [37]	At a low level of engagement (three dimensions: vigour, dedication, and absorption) distress among non-health essential workers was higher than at intermediate and high levels At a low level of sense of coherence (three dimensions: comprehensibility, manageability, and meaningfulness) distress was higher than at intermediate and high levels	35.5% of non-health workers were not provided with the means to effectively and safely carry out their activity Satisfaction at work Perception of an increase in labour conflicts during the pandemic	N/R
		Ruiz-Frutos et al. (2021) [36]	65.1% of all non-health workers had psychological distress Those non-health workers who worked away from home showed higher distress than those who worked from home	Working away from home, being women, and young age may increase the level of psychological distress	N/R
		Ruiz-Frutos et al. (2020) [44]	Risk of being infected by COVID-19 and degree of concern of being a carrier and transmitting the virus to family members, close persons, or patients were higher among those non-health workers working away from home than those working from home	Being female and having pre-existing mental health conditions were associated with higher psychological distress and fear Increased smoking and increased alcohol drinking in the past four weeks were also significant factors for fear and distress	N/R
		Bell et al. (2021) [58]	Those in healthcare and those in “other” essential work were at 71% and 59% greater risk, respectively, of moderate levels of anxiety, than those in non-essential work	Maintaining contact with family and friends, outside the bubble (via the Internet) Uncertainty around finance and employment	Increased use of alcohol

Note: N/R: not reported.

## Data Availability

Not applicable.
